# Case Report: Anthropometric profile and weight control strategies in a world boxing champion

**DOI:** 10.3389/fspor.2025.1606856

**Published:** 2025-09-02

**Authors:** Rodrigo Yáñez-Sepúlveda, Carlos Abraham Herrera-Amante, Wiliam Carvajal-Veitía, Luis Aarón Quiroga-Morales, Diego A. Bonilla, Guillermo Cortés-Roco, Nicole Aguilera-Martínez, José Francisco López-Gil

**Affiliations:** ^1^ Faculty of Education and Social Sciences, Universidad Andres Bello, Viña del Mar, Chile; ^2^ Nutritional Assessment and Nutritional Care Laboratory (LECEN), Division of Health Sciences, Tonal University Center, University of Guadalajara, Tonalá, México; ^3^ Ibero-American Network of Researchers in Applied Anthropometry (RIBA²), Almería, Spain; ^4^ Subdirectorate of Teaching and Research, Institute of Sports Medicine (IMD), Havana, Cuba; ^5^ Facultad de Ciencias de la Salud, Autonomous University of Guadalajara, Zapopan, Mexico; ^6^ Research Division, Dynamical Business & Science Society—DBSS International SAS, Bogotá, Colombia; ^7^ Grupo de Investigación NUTRAL,Facultad de Ciencias de la Nutrición y los Alimentos, Universidad CES, Medellín, Colombia; ^8^ Faculty of Life Sciences, Universidad Viña del Mar, Viña del Mar, Chile; ^9^ Facultad de Ciencias de la Salud, Universidad Católica del Maule, Talca, Chile; ^10^ School of Medicine, Universidad Espíritu Santo, Samborondón, Ecuador; ^11^ Vicerrectoría de Investigación y Postgrado, Universidad de Los Lagos, Osorno, Chile

**Keywords:** boxing, combat sports, anthropometry, body composition, somatotypes, sports nutritional sciences

## Abstract

**Purpose:**

This case study aims to examine the anthropometric profile and body composition management of a Mexican featherweight world champion during key preparatory periods leading up to the World Boxing Organization (WBO) title fight. Special emphasis is placed on the strategic role of nutritional periodization in achieving optimal body composition while preserving performance capacity in a body mass-restricted, high-performance setting.

**Methods:**

Anthropometric evaluations and octopolar bioelectrical impedance (BIA) measurements were conducted to assess body composition. The Heath and Carter somatotypes were calculated, complemented by proportionality analysis via the Phantom model of Ross and Wilson. Energy availability (EA) was estimated on the basis of fat-free mass (FFM) obtained via BIA, energy intake was recorded with the Automated Self-Administered 24-Hour (ASA24) Dietary Assessment Tool, and exercise energy expenditure was estimated through heart rate monitoring via the Hilloskorpi method.

**Results:**

The boxer presented low body fat levels (7.0% by BIA, sum of eight skinfolds = 45.0 mm) and high muscle mass percentages (53.1% by BIA and 46.6% by anthropometry). His somatotype was mesomorphic ectomorphic (1.5–2.8–5.2), with favorable body proportions, including a high relative span and ponderal index, alongside low cormic and adipose/muscular indices. Through nutritional periodization, a total body mass reduction of 6.85 kg was observed, including an acute loss of 5.35 kg (8.56%) during the final 10 days. Energy availability ranged from 35.7 kcal/kg FFM/day at the start of the preparation to 8.8 kcal/kg FFM/day in the final week before the fight.

**Conclusion:**

This case study highlights the relevance of regular monitoring of body composition through anthropometry and BIA assessment, combined with nutritional periodization, in supporting athlete preparation. Although very low energy availability was estimated during the final weeks, which should be carefully considered, the findings offer practical insights for body mass management strategies in elite boxing.

## 1 Introduction

Management of body mass is a critical factor in the performance, health, and success of athletes in combat sports, such as professional boxing (1). Despite its popularity and cultural significance, research on body mass management in professional boxing remains limited, particularly regarding its impact on both performance and health outcomes (2, 3). This gap underscores the need for evidence-based strategies tailored to the specific physiological demands imposed by different boxing weight categories.

Body composition analysis, including the assessment of anthropometric profiles, is essential for effective mass management. Recent studies have highlighted the importance of parameters such as muscle mass, fat percentage, and hydration status in optimizing athletic performance while minimizing the health risks associated with rapid body mass cutting (4–6). These factors are especially critical in body mass-restricted categories such as featherweight (126 lbs or 57.15 kg), where athletes must balance physical strength with strict mass requirements. As noted by Reale et al. (1), lighter weight divisions often demand more precise control, increasing the likelihood of acute body mass loss strategies. The most common methods of PRP used by athletes in combat sports specialties are dehydration and food restriction. It is essential to note that previous studies have demonstrated that athletes in combat sports frequently experience rapid body mass loss before the pre-fight weigh-in, followed by rapid body mass gain before competition (7, 8), which could impact their competitive success. In this regard, Baribeau et al. (7) found that, in male boxers, winners regained significantly more relative body mass than losers. Each percentage increase in body mass resulted in a 7% increase in the probability of victory in mixed martial arts and a 13% increase in boxing.

Nutritional periodization, defined as the strategic planning of dietary intake in accordance with the demands of training and competition, has emerged as a pivotal approach in managing body mass (9). In professional boxing, this methodology allows athletes to optimize body composition, supporting lean mass maintenance while mitigating the negative consequences of rapid body mass reduction. Studies suggest that nutritional periodization can lead to favorable changes in body composition while preserving performance and reducing the risks associated with extreme body mass reduction protocols (1, 10, 11).

Of particular interest is the impact of body mass management on the broader anthropometric profile. Beyond influencing physical performance, a boxer's anthropometric characteristics also shape tactical behavior and fighting style, with specific body compositions often favoring certain approaches in the ring (12, 13). Despite this, little research has directly addressed how mass management strategies affect anthropometric profiles in elite-level boxers.

Existing strategies used by combat athletes to manage body mass are diverse (1). In both professional and amateur boxing, however, few studies have examined their effect on the full anthropometric profile, focusing instead on more general body composition metrics (5, 6, 14, 15). Reale et al. (1) emphasize the importance of tailoring such strategies to cultural and contextual factors to optimize performance while safeguarding athlete health.

Furthermore, professional boxing is regulated by organizations such as the World Boxing Organization (WBO), the World Boxing Council (WBC), and the International Boxing Federation (IBF), each of which enforces strict guidelines for weight classifications. While these governing bodies vary in their organizational structures and criteria for title contention, they all reinforce the significance of rigorous and consistent body mass control (8). Understanding this regulatory landscape provides important context for evaluating the real-world application of body mass management strategies in elite competition.

The uniqueness of this case study lies in its focus on the anthropometric profile and body composition management of a Mexican world featherweight champion during the key preparatory periods for winning the WBO title in the 126-pound division. By concentrating on a single elite athlete, this study contributes to the growing but still limited body of literature on professional boxing and offers applied insights into how structured mass management (particularly though nutritional periodization) can support both health and performance at the highest competitive level.

The following research question guides this case study: How does body mass management, through a nutritional periodization approach, influence body composition and the anthropometric profile of an elite professional boxer during preparation for a world championship bout?

Accordingly, this study aims to examine the anthropometric profile and body composition management of a Mexican featherweight world champion during key preparatory periods leading up to the WBO title fight. Special emphasis is placed on the strategic role of nutritional periodization in achieving optimal body composition while preserving performance capacity in a body mass-restricted, high-performance setting.

## 2 Methods

### 2.1 Athlete and case study background

The boxer analyzed in this case study was Rafael Espinoza, WBO world champion in the “featherweight” division. The featherweight category encompasses fighters weighing more than 55.338 kg (122 lbs) and less than 57.152 kg (126 lbs). His professional record was 25 fights undefeated, with 27 wins (23 by KO and 4 by decision), with no losses, at the time of the study.

### 2.2 Interventions and assessments

**Figure 1:**
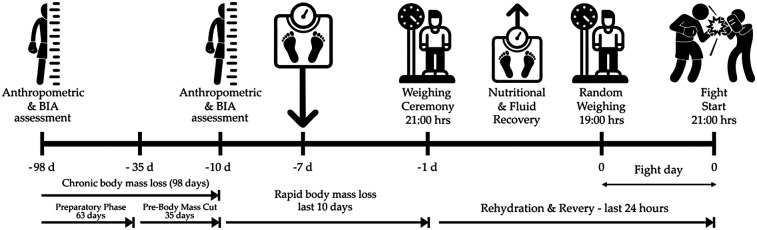
Assessment of body mass and body composition at key moments during the boxer's preparation, from 98 days before to the day of the fight. The random weighing conducted on the day of the fight was scheduled by the organizing committee.

Interventions and assessments were conducted at key points within the four phases of athlete preparation (Figure 1). The preparatory phase began on September 2, 2023, 98 days earlier. The body mass reduction phase began 35 days earlier. The rapid body mass loss phase began 10 days earlier. Finally, the rehydration and recovery phases occurred 24 h earlier. The bout took place on December 8, 2023. In all phases, body mass fluctuation and energy availability (EA) were assessed.

#### 2.2.1 Anthropometric assessment

Anthropometric assesment were performed following the international standards established by the International Society for the Advancement of Kinanthropometry (ISAK) for the assessment of 43 anthropometric variables, which included basic measurements, skinfolds, girths, breadths, and lengths (16). The measurements were performed by a certified level 3 (ISAK L3) anthropometrist.

Instruments such as a digital scale accurate to 50 g for determining body mass (SECA® 874, Hamburg, Germany); a stadiometer accurate to 1 mm for height and sitting height (SECA® 217, Hamburg, Germany); and a skinfold caliper accurate to 0. 2 mm for measuring skinfolds (Harpenden, British Indicators, Crymych, UK); a flexible, nonelastic metal tape measure accurate to 1 mm for measuring girths (SmartMet Kinanthropometric Assessment®, Jalisco, Mexico); and a segmentometer and bone caliper accurate to 1 mm for measuring lengths and breadths (SmartMet Kinanthropometric Assessment®, Jalisco, Mexico). All instruments were calibrated prior to the assessment to minimize measurement errors.

All anthropometric variables were measured at least twice. A third measurement was taken if the difference between the first and second measurements exceeded 5% for skinfolds or 1% for any other variable. The mean value was used for data analysis when two measurements were taken. The median value was used for data analysis when three measurements were taken (17). The intra-evaluator technical error of measurement (TEM) was calculated according to Pederson & Gore protocol (18). In this study, the TEM was set at 3.93% for the skinfolds and 0.91% for the rest of the variables.

Body composition was estimated via the five-way fractionation method of body mass (19). Additionally, fat mass was estimated using the Yuhasz (20), Sloan (21), Wilmore and Behnke (22) and Durnin and Womersley (23) equations. The somatotype was determined via the Heath-Carter anthropometric method (24). The raw scores of the measurements were converted into Z scores using the phantom values presented by Ross and Wilson (25). Finally, the sum of six and eight skinfolds (∑6SKF and ∑8SKF, respectively), adipose-muscular ratio; biacromial-biiliocristal breadth index, height-to-biacromial breadth index, height-to-biiliocristal breadth index, muscle-to-bone ratio, relative arm span index, and waist-to-height index were derived from raw anthropometric data.

#### 2.2.2 Bioelectrical impedance analysis

Body composition was assessed using bioelectrical impedance analysis (BIA) with an InBody® 370S analyzer. This device estimates fat-free mass (FFM), fat mass, and other body composition parameters based on segmental impedance measurements.

To ensure measurement consistency and minimize variability, assessments were conducted under standardized conditions, in accordance with best practice guidelines for BIA use (26, 27). All measurements were performed during the morning hours, in a fasted and rested state, prior to any training session, and by an ISAK-certified technician, which reduced intra-operator variability and enhanced the reproducibility of the results.

#### 2.2.3 Nutritional intervention strategy

The nutritional intervention strategy for body mass loss and management was developed based on previous recommendations (1, 28) and was structured into four progressive phases with distinct objectives and methods: (i) Preparatory Phase, focused on optimizing body composition through maximal fat mass reduction and promoting muscle mass gain; (ii) Body mass reduction phase, aimed at initiating a controlled reduction of total body mass while prioritizing the preservation of lean tissue, mainly through moderate-to-high energy restriction; (iii) Rapid Body Mass Loss Phase, targeting acute body mass reduction through three strategies: (1) continuation of energy restriction; (2) gradual fluid restriction; and (3) use of thermal clothing during training sessions. These strategies were applied during the final seven days before competition; and (iv) Rehydration and Recovery Phase, intended to restore the athlete's hydration status after weigh-in through monitored *ad libitum* fluid intake. All fluid intake was weighed and recorded to ensure accuracy.

Macronutrient intake was periodized according to training demands and the specific goals of each phase: carbohydrate intake ranged from 3–6 g/kg/day, protein from 1.6–2.8 g/kg/day, and dietary fat accounted for 25%–30% of total energy intake (0.8–1.4 g/kg/day). Hydration protocols were individualized to maintain fluid balance and optimize performance, with daily water intake prescribed at 1 ml per kilocalorie consumed. To monitor adherence, fluid intake was generally recorded via self-reported surveys throughout most phases. However, during the final two preparation phases, fluid intake was measured more precisely by weighing consumed liquids. All phases were supervised by a certified sports nutritionist and were adjusted weekly based on training load, body composition, and recovery indicators.

#### 2.2.4 Training monitoring and volume recording

Training load and total weekly training hours were monitored using a Polar H10 heart rate monitor in conjunction with the Polar Beat mobile application. This system allowed for precise, real-time tracking of the duration and intensity of each training session.

All data were automatically recorded and stored in the athlete's personal training log via the Polar Flow ecosystem, supervised directly by the coaching and support team.

The training program consisted of two daily sessions (morning and afternoon), six days per week, totaling 12 sessions per week. Monitoring covered the full duration of the intervention protocol, including preparatory and pre-competition phases.

#### 2.2.5 Energy availability assessment

Energy availability was estimated through three key parameters. First, fat-free mass (FFM) was measured via BIA using an InBody Model 370S analyzer. Second, energy intake was assessed by a certified sports nutritionist using the Automated Self-Administered 24-Hour (ASA24) Dietary Assessment Tool. Finally, energy expenditure during exercise was estimated using the Hilloskorpi method (Model 2), which is based on exercise heart rate (29). For this purpose, a Polar H10 heart rate monitor and the Polar Beat mobile application were used to calculate energy expenditure during exercise based on heart rate. Energy availability was expressed in kilocalories per kilogram of fat-free mass per day (kcal/kg FFM/day).

EA was calculated exclusively on the days when FFM was assessed via BIA. Consequently, the frequency of EA estimation aligned with the scheduled body composition assessments conducted throughout the training process. Specifically, BIA measurements, and thus EA calculations, were performed on days 98 (Preparatory Phase), 35 (Body mass reduction phase), and 15 and 7 (Rapid Body Mass Loss Phase) prior to the competition.

#### 2.2.6 Hand grip strength assessment

Hand grip strength was assessed using isometric dynamometry with a TAKEI® dynamometer (model SMEDLEY III T-16K). Three trials were performed on each hand, alternating sides, following the Southampton protocol (30), which is recommended in recent literature (31). The participant was seated on the same chair for all measurements, with the shoulder adducted and neutrally rotated, the elbow flexed at 90°, and the forearm in a neutral position resting on the armrest of the chair. The wrist was positioned just over the edge of the armrest in a neutral position with the thumb facing upwards. The feet remained flat on the floor throughout the assessment. Verbal encouragement was provided during each trial, following the standardized instruction: “Squeeze as hard as you can for as long as you can, until I say stop… squeeze, squeeze, squeeze… stop.” The highest value obtained from all six trials (three per hand) was recorded as the final grip strength score.

### 2.3 Ethical considerations

The athlete read, approved, and provided written informed consent for the publication of this case study, including the explicit use of his name and identity. All procedures adhered to the ethical principles of the Declaration of Helsinki for research involving human subjects (32). This study was developed in accordance with the CARE guidelines for Case Reports (33), and the protocol was approved by the Biosafety, Research, and Ethics Committees of the University of Guadalajara (CEI062020-01).

### 2.4 Statistical analysis

The mean was used to describe the variables. Variations in the variables were analyzed on the basis of the delta value of change (***Δ***). The analyses were performed with GraphPad® version 2.13.1 for Windows. ISAK Metry® software was also used for the analysis of body composition and proportionality values.

## 3 Results

### 3.1 Anthropometric profile and changes during the preparatory and body mass reduction phase

Table 1 shows the results of the anthropometric evaluations performed at two points during the athlete's preparation: the preparatory phase (September 2, 2023) and the phase prior to the body mass reduction (November 29, 2023). Body mass decreased by 2.3% over this nearly three-month period. For body composition, as assessed by five-way fractionation, adipose tissue mass decreased by 16.9% (from 17.2–14.3 kg), whereas muscle mass increased by 6.3% (from 28.8–30.6 kg).

**Table 1:** Anthropometric and body composition profile of the boxer.

Variable		Preparatory phase 02/09/2023	Body mass reduction phase 29/11/2023	*Δ* (%)
Basics	Body mass (kg)	64.0	62.5	−2.3
	Stretch stature (cm)	183.1	183.1	0.0
	Sitting height (cm)	91.5	91.5	0.0
	Arm Span (cm)	189.2	188.8	−0.2
Five-way fractionation of body mass (Ross & Kerr, 1991)	Adipose mass (kg)	17.2	14.3	−16.9
	Adipose (%)	25.9	21.8	−15.7
	Muscle mass (kg)	28.8	30.6	+6.3
	Muscle (%)	43.2	46.6	+7.9
	Bone mass (kg)	7.9	7.9	0.0
	Bone (%)	11.9	12.0	+1.2
	Residual mass (kg)	8.9	9.0	+1.8
	Residual (%)	13.3	13.8	+3.4
	Skin mass (kg)	3.8	3.8	0.0
	Skin (%)	5.8	5.8	0.0
Body fat—Two-way fractionation of body mass	Durnin y Womersley, 1974 (%)	13.6	9.8	−28.2
	Durnin y Womersley, 1974 (kg)	8.7	6.1	−29.9
	Sloan, 1962 (%)	8.2	6.2	−24.9
	Sloan, 1962 (kg)	5.3	3.9	−26.7
	Wilmore & Behnke, 1969 (%)	12.9	9.9	−23.4
	Wilmore & Behnke, 1969 (kg)	8.2	6.2	−25.2
	Yuhasz, 1962 (%)	10.6	8.8	−17.1
	Yuhasz, 1962 (kg)	6.8	5.5	−19.0
∑ Skinfolds	∑6 SF^a^	50.0	35.0	−30.0
	∑8 SF^b^	64.0	45.0	−29.7
Somatotype	Endomorphy	2.1	1.5	−26.9
	Mesomorphy	2.5	2.8	+10.6
	Ectomorphy	5.0	5.2	+5.3
	X	2.9	3.7	+28.6
	Y	−2.0	−1.2	−41.0
	SAD	0.7	0.0	−100.0
Indices	BMI (kg/m^2^)	19.1	18.6	−2.3
	WHI	0.4	0.4	0.0
	HBBI	22.7	22.7	0.0
	HBiiL	7.0	7.0	0.0
	BBI	1.6	1.6	0.0
	MBR	3.6	3.9	6.7
	AMR	0.6	0.5	−21.9
	Crural	1.0	1.0	0.0
	Cormic	50.0	50.0	0.0
	Relative Arm Span Index	103.2	103.2	0.0
	Relative length of the upper limb	45.6	45.6	0.0
	Braquial	72.4	72.4	0.0
	Thoracic	62.5	61.6	−1.5
BIA	Skeletal Muscle Mass (kg)	33.0	32.9	−0.3
	Body fat (%)	8.3	7.0	−15.7
	Body fat (kg)	5.3	4.4	−17.0
	Fat Free Mass (kg)	58.7	58.1	−1.0
	Total body water (L)	43.0	42.5	−1.2
	Protein (kg)	11.6	11.6	0
	Minerals (kg)	4	4	0
	InBody Score	70.0	69.0	−1.4

Δ, change (%) from T_1_ to T_0_; ∑6SKF, sum of 6 skinfolds; ∑8SKF, sum of 8 skinfolds; AMR, adipose-muscular ratio; BBI, biacromial-biiliocristal breadth index; BIA, bioelectrical analysis impedance; BMI, body mass index; HBBI, height-to-biacromial breadth index; HBii, height-to-biiliocristal breadth index; MBR, muscle-bone ratio; RASI, relative arm span index; WHR, waist-to-height index.

^a^ Sum of triceps, subscapular, supraspinal, abdominal, front thigh, and medial calf skinfold thicknesses.

^b^ Sum of triceps, subscapular, biceps, iliac crest, supraspinal, abdominal, front thigh, and medial calf skinfold thicknesses.

For the estimation of fat mass from bicompartmental methods, decreases of 29.9% (from 8.7–6.1 kg), 26.7% (from 5.3–3.9 kg), 25.2% (from 8.2–6.2 kg), and 19% (from 6.8–5.5 kg) were observed, according to the methods of Sloan (1962), Yuhasz (1962), Wilmore and Behnke (1969), and Durnin and Womersley (1974), respectively. In addition, a reduction of 21.9% was observed in the adipose-muscular ratio (AMR) index, whereas the MBR index decreased by 6.7%. In general, the other indices reflecting skeletal characteristics remained relatively stable. However, body proportions reflected a high relative wingspan (103.2 cm), accompanied by a low cormic index.

**Figure 2:**
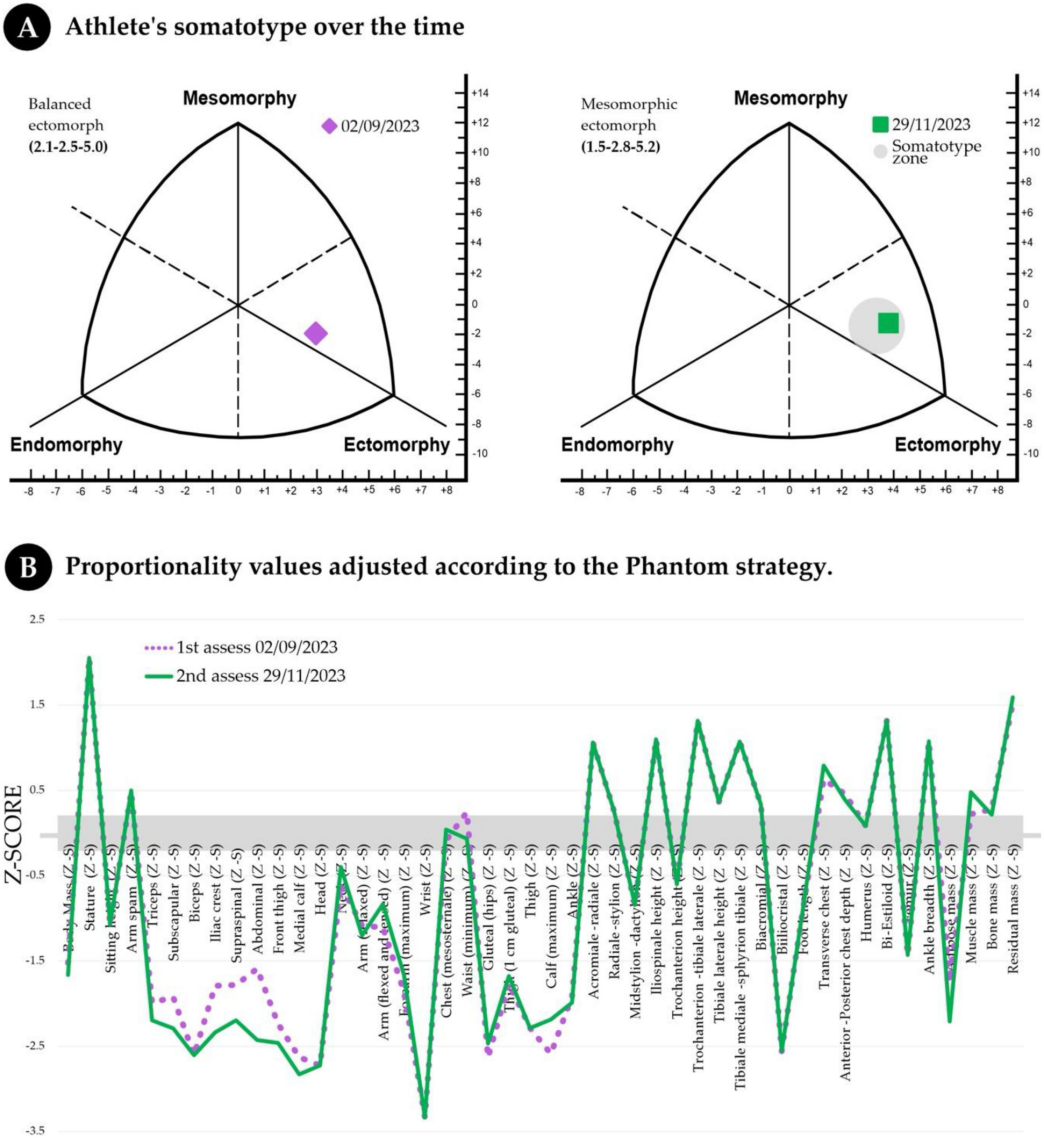
Body mass fluctuation, somatotype and human proportionality based on anthropometric variables (anthropometric profile) at key moments in the boxer's preparation (graphs **A, B**).

Figure 2A shows the somatotype at the two anthropometric evaluation points. At the first evaluation (01/06/2023), the athlete presented a balanced ectomorph somatotype (2.1–2.5–5.0), which changed five months later to a mesomorphic ectomorph somatotype (1.5–2.8–5.2). During this transition, the athlete substantially reduced relative adiposity by 26.9% and, although in a smaller proportion, exhibited increases in relative musculoskeletal development (10.6%) and relative linearity (5.3%).

Figure 2B presents a graphical comparison of the *Z* values evaluated in each period for each anthropometric variable, as determined by the phantom method. A relatively stable proportionality profile was obtained over time, although the Z-Phantom scores of the skinfolds decreased, except for those of the biceps. On the other hand, the Z-Phantom score of the leg increased during the second measurement.

**Figure 3:**
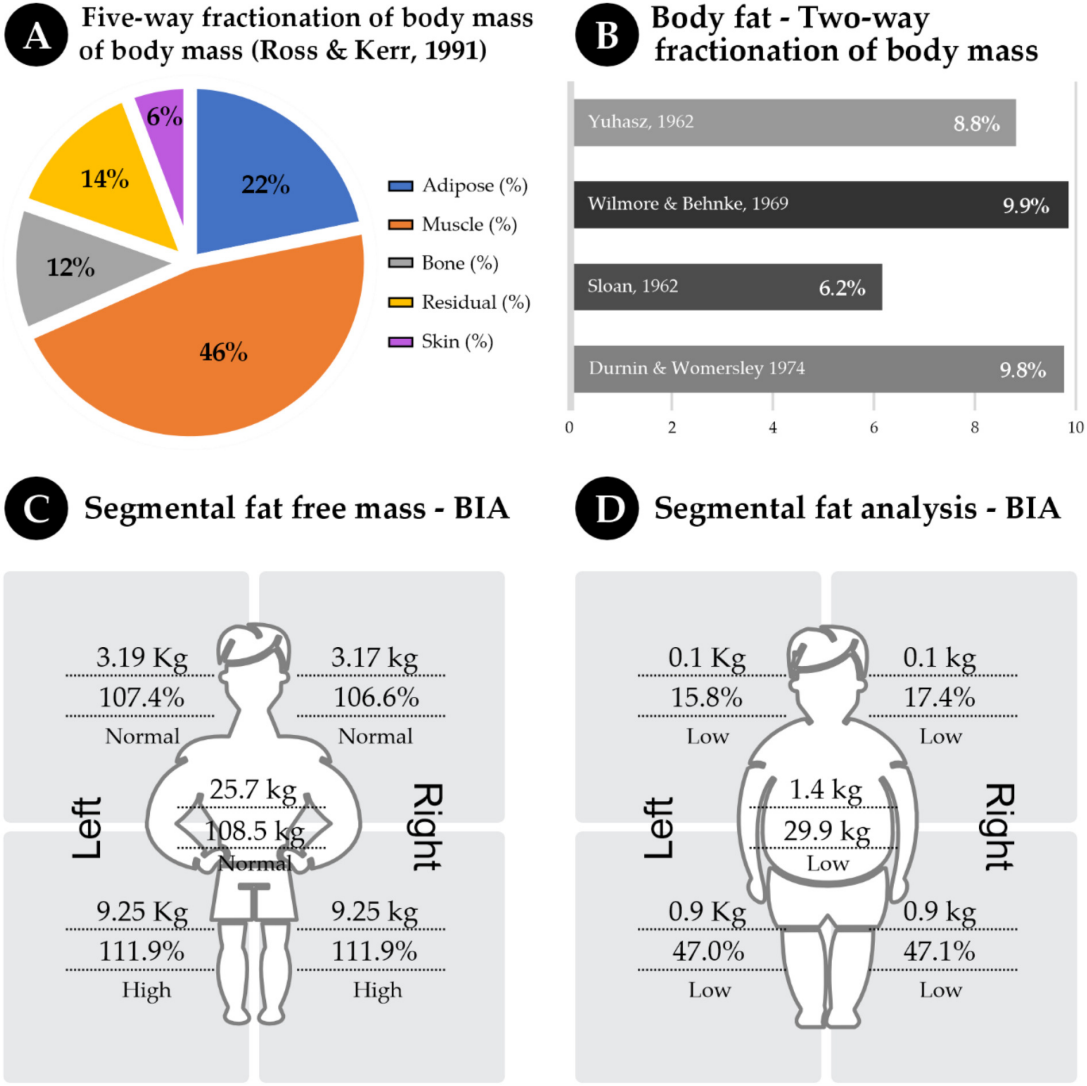
Pie chart **(A)** illustrates the five-way fractionation of body mass into adipose tissue, muscle, bone, residual mass, and skin, with skin mass representing the smallest fraction and muscle mass the largest. Bar graph **(B)** presents body fat estimates derived from two-way fractionation using different equations. Segmental fat-free mass analysis **(C)** depicts the percentage distribution across body parts, while segmental fat analysis **(D)** shows low fat percentages in a similar body diagram. All graphs **(A–D)** display data collected on 29/11/2023, ten days before the fight, during the body mass reduction phase.

In Figure 3, graphs C and D present the fractionation (C) and bicompartmental (D) estimates of body mass. Graphs E and F show the estimates obtained via segmental BIA. All the results reflect body composition characteristics at the date closest to the competition (11/29/2023), which was only 10 days from the official weigh-in.

Figures 3A-D show the fat-free mass and fat mass measured via BIA.

### 3.2 Management of body mass in the acute phase and nutritional period

**Figure 4:**
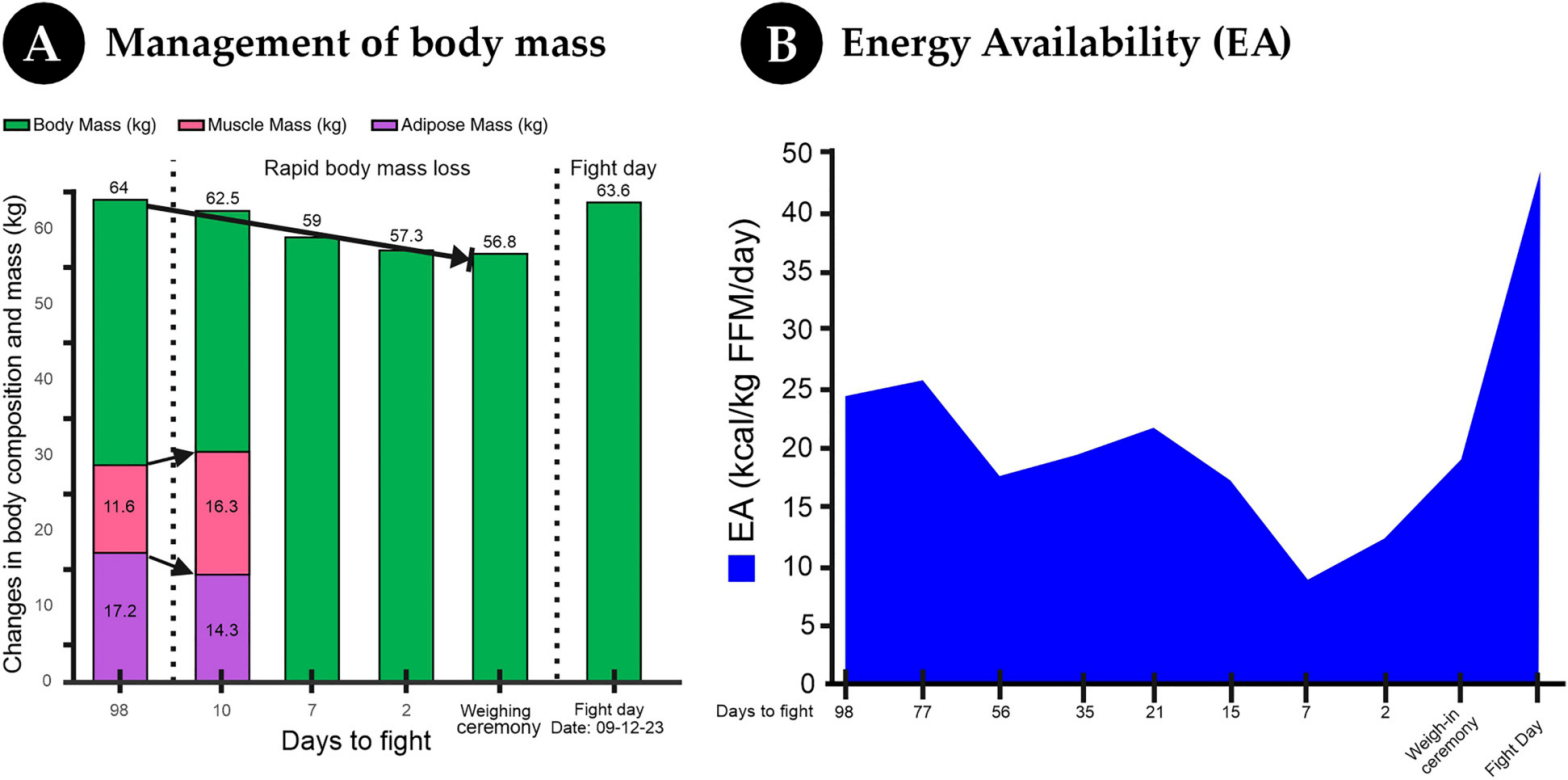
Two-part chart on body mass management and energy availability. **(A)** Bar chart showing body mass changes over the 98 days leading up to the fight. Rapid loss from 64 kg to 56.8 kg occurs before the fight, followed by an increase to 63.6 kg on fight day. **(B)** Line graph depicting energy availability fluctuations, initially around 25 kcal per kg of fat-free mass, dipping below 20, and peaking on fight day.

During the three months and seven days between 02/09/2023 and 29/11/2023, the boxer experienced a reduction in body mass from 64 kg–62.50 kg. In the final phase, ten days before the competition, he had to register an additional loss of 5.35 kg to comply with the regulatory body mass, which represented an acute reduction of 8.56% (Figure 4, Graph G).

The EA fluctuated throughout the preparation. It reached a peak of 25.7 kcal/kg FFM/day 76 days before the fight during the preparatory phase, whereas 56 days prior to the fight, it decreased to 19.4 kcal/kg FFM/day, reflecting an 18.4% decrease in energy intake.

During the body mass reduction phase, EA increased from 19.4–21.7 kcal/kg FFM/day due to a 9.81% reduction in exercise energy expenditure. In the body mass reduction phase (15 days before consumption), the initial EA was 17.2 kcal/kg FFM/day, which decreased to 8.8 kcal/kg FFM/day during the rapid loss phase, resulting in a 25% decrease in energy intake. Finally, the recovery phase (24 h prior to combat) resulted in a substantial increase in EA to 47.9 kcal/kg FFM/day (Figure 4B).

Table 2 shows the nutritional period of the athletes, which was divided into four phases.

**Table 2:** Nutritional periodization and body composition assessment at key points during preparation.

Phases	Days before fight	Body Mass (kg)	FFM (kg)	EI (kcal/day)	CHO (g/kg)	Protein (g/kg)	Fat (g/kg)	EEE (kcal/day)	EA (kcal/kg FFM/day)	ADH (ml/d)
Preparatory	98	62.8	57.5	3,052.1	6	3	1.4	1,650	24.4	3,000
	77	62.8	57.5	3,052.1	6	3	1.4	1,575	25.7	3,000
	56	62.8	57.5	2,524.6	5	2.8	1	1,515	17.6	2,500
Body mass reduction phase	35	62.5	58.6	2,512.5	5	2.8	1	1,375	19.4	2,500
	21	62.5	58.6	2,512.5	5	2.8	1	1,240	21.7	2,500
	15	62.5	58.1	2,000	4	2.2	0.8	1,000	17.2	2,000
Rapid Body Mass Loss	7	59	58.1	1,510.4	3	1.6	0.8	1,000	8.8	1,500
	2	57.3	58.1	1,466.9	3	1.6	0.8	750	12.3	500
	Weigh-in Ceremony (08/12/2023)	56.8	58.1	1,454.1	3	1.6	0.8	353	19	0
Rehydration & Recovery	Fight Day (09/12/2023)	63.6	58.1	2,785.7	5	2.8	1.4	0	47.9	6,800

FFM, fat-free mass (kg); EI, energy intake (kcal/day); EEE, exercise energy expenditure (kcal/day); EA, energy availability (kcal/kg FFM/day); ADH, average daily hydration (ml/d).

EEE was estimated using the Hilloskorpy method. FFM was determined by Bioelectrical Impedance Analysis (BIA). EI was obtained using the ASA24 tool. EA was calculated following the methodology proposed by Loucks, A. B. (2007) using the equation: EA = (Energy Intake—Exercise Energy Expenditure)/Fat-Free Mass.

In the preparatory phase, during the three evaluation periods, body mass and lean mass remained constant. Total intake, macronutrient intake and exercise energy expenditure decreased by 17.3%, 16.6% and 8.18%, respectively, as did EA (27.86%) and average water intake (17.3%) at the end of the phase on day 56.

In the second body mass reduction phase, body mass, fat-free mass and daily intake remained constant, as did macronutrient intake. However, exercise energy expenditure decreased by 9.81% between day 35 and body mass reduction phase day 21. The AD increased by 9.4%, whereas the average daily water intake remained constant.

In the third rapid loss phase, body mass decreased by 9.12% between day 15 and the day of official weighing. FFM remained at 58.1 kg, whereas total intake decreased by 27.3%. Carbohydrates (CHO) intake was 4 g/kg 15 days prior to the fight and decreased to 3 g/kg, remaining stable during the last 7 days until the weigh-in ceremony. The protein intake was 2.2 g/kg; 15 days before the bout, it decreased to 1.6 g/kg and remained stable until the weight-in ceremony, whereas the fat intake remained constant at 0.8 g/kg. The EA values fluctuated between 17.2, 8.8, 12.3 and 19 kcal/kg FFM/day, respectively. The average daily water intake decreased by 66.8% two days before weighing, reaching 0 (no water intake) on the day of the weighing ceremony.

In the prefight rehydration phase (last phase), body mass increased by 9.12%, fat-free mass remained constant, and total intake increased by 47.72% compared with that in the previous phase. The average daily water intake increased from 0 ml/d (on the day of the weigh-in ceremony) to 6,800 ml/d in this last phase.

### 3.3 Hand grip strength assessment changes

Hand grip strength was assessed during three distinct phases of preparation: the Preparatory phase (98 days before competition), the Body mass reduction phase (35 days before), and the Rapid Body Mass Loss phase (2 days before). In the right arm, grip strength decreased from 46.0 kg–44.8 kg between the Preparatory and Body mass reduction phase (a decrease of 1.2 kg), and then further declined to 42.7 kg before the competition (a total decrease of 3.3 kg). Similarly, the left arm showed an initial drop from 45.6 kg–42.5 kg (−3.1 kg) followed by a slight recovery to 43.0 kg in the final measurement.

## 4 Other results

For training, the weekly time dedicated to sports practice decreased progressively from 20.6–10 h, with a fixed rest day per week.

## 5 Discussion

This is one of the first studies to present the anthropometric and body composition profile of a world featherweight champion during four key periods of his preparation before winning the WBO title in the 126-pound category. The boxer exhibited a low body fat percentage according to different assessment methods, with closely aligned values using BIA and the bicompartmental model and elite athlete references from previous studies (34), but notably higher values when applying the pentacompartimental body mass fractionation method. Muscle mass estimates varied depending on the method, with higher values obtained through BIA compared to anthropometry. The somatotype was classified as a balanced ectomorph, reflecting predominantly linear characteristics. The athlete also showed high relative arm span and ponderal index, along with low values in the formic and adipose/muscular indices. Overall, the morphological profile indicates a body composition well suited for high-demand sports such as boxing (34).

Although the effects of dehydration were not measured in our study, some reviewed studies provide strong evidence of the negative effects of rapid and extreme body mass loss on the physical performance and health of combat sports athletes. Barley et al. (35) demonstrated that acute dehydration of 4.8% of body mass significantly impairs physical performance up to 24 h later, questioning the common practice of pre-competition body mass reduction phase. In a complementary manner, Zubac et al. (36) found that even a more moderate body mass loss (3%) decreases neuromuscular performance in elite boxers, suggesting that there is no safe threshold for this practice without compromising athletic ability. Taken together, these findings underscore the need for stricter regulations and safer body mass management strategies in combat sports to preserve both the performance and the short- and long-term health of athletes.

There is limited evidence on the anthropometric and somatotype characteristics of featherweight boxers to contrast the results of the variables evaluated. Rather, there is scarce evidence in boxers differentiating weight categories and, in general, in combat sports (4, 34–38). Among the few studies performed in boxers of this competitive category, Morton et al. (14) carried out a case study in a boxer whose body mass decreased over 12 weeks, leaving his lipid mass at 7% from a starting point of 12.1%. On the other hand, Carvajal et al. (39) reported values of 37.6 mm in the sum of six panicles (7.5% lipid fat) in Olympic boxers in the 56 kg category, who had an average height of 169.5 cm and a predominantly ectomorphic mesomorph somatotype (1.6-4.4-3.0) at the time of the study.

The somatotype of this athlete may be atypical compared to the somatotype of Cuban Olympic boxers, which are usually ectomorphic mesomorphs in almost all categories below 75 kg. In the 1980s, Rodriguez et al. (40) characterized a population of boxers and concluded that the somatotype is closely related to the fighting style; a mesomorphic ectomorphic somatotype and arms of large wingspan could condition a fighting style from a long distance, as occurs in this boxer, who also has a height of 183.1 cm, which is much greater than the average height of Cuban Olympic boxers in the same competitive category (37).

The results obtained by Tshibangu (12) support those certain somatotypes, more or less ectomorphic, influence sports performance. This same author studied a population of 98 of the 100 best male professional boxers in the world ranked until 2013 and showed that ectomorphic values higher than 2.5 increase their percentage of victories by knockout (KO) in a polynomial way. In the case study, the boxer has an ectomorphy value of 5.2, which coincidentally equates to a KO win percentage of 84% as this boxer's record.

On the other hand, rapid body mass loss consists of a protocol that allows competitive division to be reached a few days before the weigh-in ceremony (11). If strictly necessary, rapid body mass loss may be the ideal option to reduce body mass, but it should not exceed 5% only if the time between weight-in and competition is sufficient to refeed and rehydrate (more than 3 h) (41). In this study, during the acute phase, the boxer had to record an additional loss of 5.35 kg to meet the regulatory body mass, which represented an acute reduction of 8.56%. One method of reducing total body water is fluid restriction relative to normal daily losses. Body water reduction can be derived from extracellular and intracellular stores, i.e., free and bound water (glycogen stores and the intestinal space) (42). The athlete decreased three times the average daily hydration within his nutritional period during the period of rapid/acute body mass loss. Elite boxers reportedly experience significant hypohydration, with a body mass loss of 5% before competition (8). On the other hand, with respect to body mass recovery, athletes with rapid losses before competition quickly regain their previous body mass after the end of the competition period (43). In the present study, in the rapid body mass loss phase (competition day), the athlete exceeded the body mass of the beginning of the body mass loss phase (62.5 kg), reaching a final body mass of 63.6 kg.

At 12 weeks and 5 days until October 31, 10 days before the competition, the athlete lost only 2.3% of his body mass; 10 days after the competition, his body mass decreased to 8.56% in the acute phase. Combat athletes in light categories have very discrete body fat values (36). As lean mass is the limiting factor, many prefer to remain during the chronic phase with stable values of body mass, especially when they usually should not lose more than 10% of their body mass, and then reduce in the acute phase, as occurred in this case, with a loss of 8.56%. This strategy has been documented mainly in combat sports, such as Olympic wrestling (8, 10, 44, 45). These results are considered positive regarding the nutritional periodization objective of achieving optimal body mass according to the athlete's category. However, from a health management point of view, previous studies have shown the harmful effects of extreme weight cutting on mixed martial arts athletes, which represent a call for action by governing bodies to safeguard the welfare of mixed martial arts athletes (46). We suggest considering this when athletes use dehydration to lose weight 24 h before the competition.

Nutritional periodization proved an effective strategy for body mass management in boxers, allowing gradual adjustments in energy and macronutrient intake that favour the preservation of fat-free mass even in critical phases of rapid body mass loss. These results are in line with the findings of Langan-Evans et al. (47) who, in a case study, showed that fluctuating EA, with a mean value of 20 kcal/kg FFM/day over seven weeks, reduced body mass and fat without triggering alterations in physiological systems linked to the Male Athlete Triad and Relative Energy Deficiency in Sport (RED-S) syndrome. However, they also noted that more aggressive and sustained restriction, less than 10 kcal/kg FFM/day for five consecutive days, did produce adverse consequences associated with these syndromes. These findings underscore the importance of careful nutritional planning that considers reaching a competitive weight and preserving health and performance throughout the process. In the rehydration and recovery phases, a significant increase in caloric intake and hydration was subsequently implemented, increasing energy availability and facilitating the recovery of initial body mass. These results align with studies highlighting the importance of specific nutritional strategies to minimise the risks associated with extreme body mass management in sports competing in body mass categories (10, 11).

In combat sports, glycogen depletion is used to decrease body mass (42), and one of the methods leading to lower glycogen stores is a low-carbohydrate diet combined with a regular training program (11). Recommendations suggest following a low-carbohydrate diet (2–3 g/kg/d) to avoid the restoration of muscle glycogen stores after their depletion by skill training and/or additional cardiovascular activity (48). In our study, within the nutritional period, carbohydrate intake decreased from 6 g/kg in the preparatory phase to 3 g/kg in the rapid loss phase, with a 25% decrease in exercise energy expenditure. On the other hand, in boxing, the quality of muscle strength in both the upper and lower extremities can affect an athlete's success (41). In this context, antecedents collected from a systematic review with meta-analysis of the published literature on rapid loss of body mass in Olympic combat sports indicate that processes of rapid loss of up to 5% of body mass could be beneficial for athletes since they do not negatively influence their performance, considering the aspects of power and strength (49). The determining factor for decreased performance would be hydration or food after the weigh in ceremony and before the fight (50). In this study, in the rehydration phase and on the day of the fight, the boxer underwent a rigorous rehydration process that reached 6,800 ml/d, resulting in an increase in caloric intake and a 40% increase in the consumption of macronutrients, mainly carbohydrates.

Handgrip strength is a recognized neuromuscular marker in combat sports, particularly boxing, due to its influence on punching power, clinch control, and upper-body function. Studies indicate that elite boxers typically have dominant-hand grip strength values between 40 and 45 kg (51), while young boxers show significantly lower values (mean 34.80, IQR 38.45–30.10) (46). In our case study, the athlete showed mean values of 44.5 kg in the right hand and 43.7 kg in the left—both within the elite reference range. During the weight-reduction process, right-hand strength declined to 42.7 kg and left-hand strength stabilized around 43.0 kg, yet remained close to the lower end of elite values. These results suggest that although grip strength decreased over the time, it remained within competitive standards and did not appear to compromise neuromuscular performance. Future longitudinal studies or larger cohorts could leverage handgrip strength measurements to monitor functional preservation across weight management cycles.

About EA, a previous case study in a taekwondo athlete reported effects of low EA on health and performance indices during five consecutive days of EA <10 kcal/kg FFM/day, which resulted in consequences associated with low EA, as evidenced by disruptions in hypothalamic‒pituitary‒gonadal axis hormones, which were rapidly reversed during the rebound hyperphagic response that occurred between 1 and 7 days after competition (47). Regarding this approach, it has recently been suggested that an energy availability of 20 kcal/kg FFM/day is problematic for health (47). In our study, although there were fluctuations in the EA during the different periods, the lowest EA was reached one week before the official weigh-in; however, it increased 53% on the day of the weigh-in, mainly due to a decrease in the energy expenditure of exercise, without a history of suffering from these alterations, and subsequently reached a maximum value of 47.9 kcal/kg FFM/day on the day of the fight. Although this finding showed a rapid recovery in EA, it remained below 20 kcal/kg FFM/day, thus highlighting the risks associated with energy restrictions in combat sports and demonstrating the need to continually monitor health parameters for possible manifestations of the male athlete triad.

The data presented in the nutritional planning of the case study show a progressive approach to body mass reduction and its management over an extended period (98 days, with 4 phases, including a specific phase), minimizing the use of extreme dehydration methods that could compromise the athlete's health. This finding is in line with what has been reported in the literature, which indicates that dehydration practices, both active and passive, can cause severe renal damage and even become fatal in extreme circumstances (52). It is worth noting that the average daily hydration level does not directly indicate dehydration. Therefore, we emphasise the importance of objectively assessing dehydration during the process, considering that acute dehydration of 4.8% of body mass results in a reduction in physical performance 3 and 24 h after dehydration (34), once again highlighting the importance of that caution should be exercised when athletes use dehydration to lose weight 24 h before competition.

On the other hand, although this case presents a detailed longitudinal analysis of a boxer during key periods of preparation, its focus on a single subject limits the generalisation of the results to other athletes or combat disciplines. It would be desirable to conduct complementary studies with larger samples and varied contexts to validate the patterns observed. Despite these limitations, the longitudinal approach contributes to a detailed understanding of the dynamics between anthropometry and nutrition in elite boxers. However, we acknowledge a limitation in the lack of objective testing of the athlete's health status, considering the evidence from previous studies that highlights the health and performance risks in combat sports athletes competing in weight categories.

### 5.1 Implications for coaches and practitioners

This study highlights the importance of integrating advanced nutritional periodization for elite boxers, optimizing safe methods of body mass reduction. The information presented emphasises the importance of rehydration and energy recovery after weihing, critical aspects that require specific protocols to maximise competitive performance. In addition, the anthropometric profile of the boxer, with low adiposity, high muscle mass and favourable body proportions, reinforces the need to design personalised programs that integrate the athlete's physiological demands and competitive goals. Strategies include careful carbohydrate intake management and tailored nutritional planning, leveraging approaches based on previous studies that seek to optimize glycogen stores and ensure competitive success (22, 53–55). This holistic approach allows us not only to optimize performance but also to strengthen the connection between anthropometric and body composition characteristics and individual fighting styles. However, as we pointed out earlier, we emphasise the importance of incorporating objective assessments into the process to monitor the athlete's health and dehydration status.

## 6 Conclusions

The results of this study highlight the relationship between an athlete's anthropometric profile and body composition profile using BIA and their competitive success. The relevant characteristics were low adiposity, high muscle mass, and adequate body proportions. Progressive body mass planning strategies using a nutritional periodisation strategy and constant monitoring of body composition may have contributed to the athlete's competitive success. This analysis reinforces the importance of implementing personalised and safe approaches that balance physiological demands with competitive demands, providing boxing coaches and athletes with a practical application that manages nutritional preparation and body mass control in high-level combat sports, assuming that the control of health parameters during the process is crucial to ensuring success in the process.

## Data Availability

The raw data supporting the conclusions of this article will be made available by the authors, without undue reservation.
